# The Impact of the Integration of Urban and Rural Medical Insurance on Migrant Workers' Overwork: Evidence From China

**DOI:** 10.3389/fpubh.2022.934524

**Published:** 2022-07-01

**Authors:** Zengxin Xue, Bowei Li

**Affiliations:** ^1^School of Humanities and Law, Northeastern University, Shengyang, China; ^2^College of Economics and Management, Zhejiang A&F University, Hangzhou, China; ^3^Research Academy for Rural Revitalization of Zhejiang Province, Zhejiang A&F University, Hangzhou, China; ^4^Institute of Ecological Civilization, Zhejiang A&F University, Hangzhou, China

**Keywords:** integration of urban and rural medical insurance, overwork, migrant workers, health rights, double difference method

## Abstract

In recent years, the problem of migrant workers' excessive labor has attracted much attention. The implementation of the integration policy of urban and rural medical insurance has broken the urban-rural dual division system. While improving migrant workers' health and sense of social integration, can they effectively alleviate their overwork? Based on the panel data of China Labor Dynamics Survey (CLDS) in 2016 and 2018, this paper empirically analyzes the impact of the integration of urban and rural medical insurance on migrant workers' overwork by using the differential difference model (DID). The research shows that the integration of urban and rural medical insurance can significantly alleviate the excessive labor of migrant workers; Heterogeneity analysis shows that, comparing with the new generation, the eastern region, the tertiary industry and low education level migrant workers, it is more obviously that the integration of urban and rural medical insurance alleviates the overwork of the older generation, the central and the western regions, the secondary industry and high education level migrant workers. Path analysis shows that the integration of urban and rural medical insurance will improve the social identity and health level of migrant workers, and then reduce the probability of migrant workers' overwork.

## Introduction

The rapid development of industrialization and urbanization in China has attracted a large number of surplus rural labors to transfer to cities and towns, forming a large number of migrant workers and becoming the backbone of China's urban labor market. According to the monitoring and survey report on migrant workers in 2019 issued by the National Bureau of statistics, compared with 2018, the total number of migrant workers in China increased by 0.8% to 290.77 million in 2019. However, due to the generally low level of educational human capital of migrant workers, they often engage in low-level physical labor, resulting in low time rate of return of their labor unit. In order to pursue higher income, they often pay the price of long-time excessive labor, which makes excessive labor become a common phenomenon. According to the data of the National Bureau of statistics, the average working hours of urban employees in China are 45.6 h, far exceeding the 44 h stipulated by the state, of which 42.4% are migrant workers who work more than 48 h a week (1). Many studies have shown that long-term overwork will seriously damage the physical and mental health of migrant workers, and the incidence of cardiovascular and cerebrovascular diseases, mental disorders and other diseases will increase significantly with the increase of overwork time (2). Under this realistic background, how to alleviate migrant workers' excessive labor and protect their health rights and interests in cities is not only related to the realization of the goal of “Healthy China Strategy”, but also an important policy tool to tap the potential of Chinese labor force and improve social welfare.

The classical theory of labor economics points out that the coverage of medical insurance has an important impact on workers' performance in the labor market (e.g., wage level, working time and employment status). Although, China fully implemented the new rural cooperative medical insurance (NCMS) in 2003, which aims to provide basic medical security for rural residents and improve their health status. However, affected by the long-term differentiated management of China's urban and rural social security system, China's NCMS can not provide timely medical security for migrant workers. Existing studies on the effect of NCMS on rural residents have found that NCMS with non-portability or discriminatory reimbursement policies are institutional factors that significantly hinder rural labor mobility (3). The NCMS require rural residents to participate in insurance only in the registered residence, and migrant workers can not be insured at any time with the change of the working place of migrant workers. Meanwhile, compared with local reimbursement, the reimbursement threshold of NCMS is higher and the reimbursement ratio is lower (4). There is no doubt that migrant workers engaged in non-agricultural employment in cities cannot enjoy the same medical rights and interests as urban residents even if they participate in the NCMS (5). In the absence of medical security, migrant workers can only increase their working hours in the form of preventive labor supply in order to prevent the risk of health deterioration (6).

In order to further narrow the gap between urban and rural areas and improve the treatment of medical services for rural residents, the Chinese government issued the document on integrating the basic medical insurance system for urban and rural residents in 2016. The urban and rural residents' basic medical insurance (URRBMI) will integrate the new rural cooperative medical insurance (NCMS) and urban residents' basic medical insurance (URBMI). From the perspective of policy setting, the URRBMI has increased the medical institutions that migrant workers can choose, improved the convenience of medical treatment, and solved the problem of migrant workers' medical treatment in other places. Secondly, the URRBMI insurance has significantly improved the medical insurance treatment of migrant workers, and the reimbursement proportion and reimbursement catalog have increased significantly. For example, after the implementation of the URRBMI in Beijing, the maximum reimbursement proportion of migrant workers' outpatient service has increased by 5 percentage points, the maximum reimbursement proportion of hospitalization has increased by 5–10 percentage points, and the reimbursement proportion of serious illness insurance has increased by 10 percentage points. Moreover, the types of drugs that can be reimbursed have also been expanded from the current 2,510 to more than 3,000, which will greatly reduce the medical burden of migrant workers (7). As an important factor affecting migrant workers' overwork, how will the transformation from NCMS to URRBMI affect migrant workers' overwork?

According to existing studies, there are relatively few empirical studies on the effect of URRBMI policy, especially for vulnerable migrant workers. Only a few scholars have evaluated the policy effect of URRBMI from the perspectives of improving health, improving the utilization rate of medical services, promoting social integration and social participation (8–11).However, there is no special study on the impact of URRBMI on migrant workers' overwork. Only some scholars analyze migrant workers' overwork from the perspective of medical insurance system. For example, Deng (6) from the perspective of the accessibility of migrant workers' health rights and interests, found that migrant workers participating in URBMI had 8.45% less weekly labor time than those not participating, and significantly reduced the incidence of overtime work of migrant workers. Guo (12) also finds that the overtime hours of migrant workers with medical insurance are significantly shortened. However, the above studies did not distinguish between URRBMI, urban workers' medical insurance and other forms of public medical treatment.

Based on this, with the help of the good quasi natural experiment of URRBMI, this paper attempts to explore the impact of URRBMI on migrant workers' excessive labor, in order to make a scientific evaluation on the reform effect of URRBMI in the new era, and more deeply understand the deep-seated institutional factors behind migrant workers' excessive labor behavior. The possible marginal contributions of this paper are as follows: (1) From the research perspective, this paper empirically analyzes the impact of medical insurance system arrangement on migrant workers' excessive labor with the help of the policy of URRBMI for the first time, so as to provide a more rigorous empirical basis for policy improvement. (2) From the perspective of research methods, based on the panel data of CLDS in 2016 and 2018, this paper uses the Differences-in-Differences (DID) model to better alleviate the endogenous problem caused by sample deviation, and further uses the Differential Propensity Score Matching Method (PSM-DID) to test the robustness, which greatly enhances the credibility of the conclusion. (3) From the perspective of impact mechanism, existing studies only focus on outcome variables and ignore the impact channels. This paper uses the mediating effect causal step method to identify the impact channels of URRBMI on migrant workers' overwork, so as to provide theoretical reference for alleviating the problem of migrant workers' overwork in the future.

## Theoretical Analysis: The Impact of the URRBMI on Migrant Workers' Overwork

**Figure 1:**
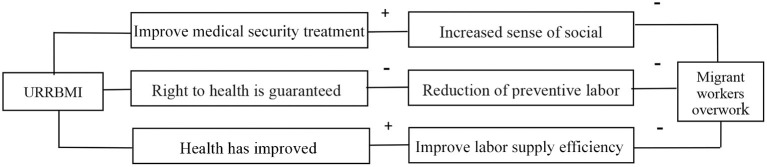
Action path of urban-rural medical insurance integration on migrant workers' overwork.

The impact mechanism of URRBMI on migrant workers' overwork mainly has the following two aspects: On the one hand, URRBMI alleviates migrant workers' overwork to a certain extent by improving their social identity. On the other hand, URRBMI can reduce excessive labor by improving the health level of migrant workers. Therefore, URRBMI has “identity effect” and “health effect” on migrant workers' excessive labor. The effect path of URRBMI on migrant workers' overwork is shown in the Figure 1.

### Identity Effect

Social identity theory was first widely used in social psychology. Akerlof and Kranton (13) first introduced this theory into the field of economics. Social identity theory holds that people always belong to a specific social group. Affected by the behavior characteristics of the group where they locate, people will change their original behavior and obey the characteristics of the group (14). Under the theory of social identity, migrant workers in China may reduce their overwork time through the imitation effect of reference points. When migrant workers have a strong sense of social identity to the inflow place, migrant workers will have strong economic integration and behavior adaptation in the inflow place, imitate local urban residents in terms of clothing, food, housing and transportation, and have behavior characteristics similar to those of local people (15, 16). The URRBMI not only improves the medical service level of migrant workers, but also significantly improves the social participation and social integration level of migrant workers at the psychological level, making migrant workers have a strong sense of urban local identity (9, 10). Sun and Wang (17) used the dynamic monitoring survey data of the floating population conducted by the National Health Commission in 2017. The empirical test found that when the floating population has urban local identity, they tend to take the local people as the reference standard, because the working hours of urban residents are relatively small. The floating population with stronger urban local identity reduces its excessive labor time through imitation effect, so as to effectively alleviate the occurrence of excessive labor. Based on the above research, it can be expected that the URRBMI can effectively reduce the probability of migrant workers' overwork by improving their sense of social integration and reducing their overwork time.

### Health Effects

The traditional labor-leisure intertemporal substitution theory holds that there is a substitution relationship between labor and time. When the wage level is high, migrant workers will increase labor supply and reduce leisure time, while when the wage level is low, migrant workers will reduce labor supply and increase leisure time. Low (18) put forward the preventive labor theory on this basis. If there is uncertainty in the future income of migrant workers, they will reduce the impact of future uncertainty by increasing the current labor time, then migrant workers will no longer allocate labor and leisure time according to the wage level. At this time, the labor-leisure intertemporal substitution theory will no longer be applicable. Based on the preventive labor theory, Chinese scholar Wang (19) made an empirical test using CHNS panel data from the perspective of health uncertainty and found that under the condition of health uncertainty, there is indeed a phenomenon of preventive labor supply for rural non-agricultural labors.

The preliminary theoretical analysis shows that the family assets of migrant workers are generally limited, and their income level is low, so they can only meet their living needs through labor income. Before the implementation of URRBMI, migrant workers could not enjoy the high-quality medical services in the inflow area. Due to the lack of necessary financial tools, it was difficult to realize the intertemporal substitution of labor force. At this time, the marginal effect of consumption tended to be infinite (6). In the case that they cannot survive without work, although the health of migrant workers is damaged by long-term and high-intensity labor, in order to meet the needs of survival, they have to increase their working hours in the form of preventive labor supply to prevent the impact of health risks. However, after the implementation of the policy of URRBMI, the health rights and interests of migrant workers have been protected, and their motivation of preventive labor supply has been weakened (20). Migrant workers allocate rest time across periods to ensure the equal marginal utility of consumption in different periods (19). From this perspective, the implementation of URRBMI provides a higher level of medical security for the health of migrant workers, and can help migrant workers optimize their intertemporal labor resource allocation and reduce labor intensity.

On the other hand, Grossman's theory holds that medical care is one of the important determinants affecting health. Consumers can increase their investment in health capital by buying medical services, while medical insurance can increase its investment in health capital by reducing the price of medical services (21). The implementation of URRBMI policy has further improved the reimbursement ratio and expanded the scope of medical treatment for migrant workers. That is, the change of the system makes the price of medical services for migrant workers drop more, so migrant workers increase spending on health, improve the medical service utilization, reduce the likelihood that migrant workers will not see a doctor if they fall ill, thus affecting health level and improve its labor yield per unit time, so that migrant workers do not need to increase their working hours to increase their economic income (22).

To sum up, the URRBMI can alleviate their overwork whether by improving the social identity of migrant workers or improving the health status of migrant workers. Based on this, we can draw the following inferences and assumptions:

*Hypothesis 1*: URRBMI can significantly alleviate the Overwork of migrant workers.
*Hypothesis 2*: URRBMI reduces the probability of migrant workers' overwork by improving their social identity and health level.

## Empirical Research: The Impact of the URRBMI on Migrant Workers' Overwork

### Data Sources

The data selected in this study are from China labor force dynamic survey (CLDS) in 2016 and 2018. The survey is a large-scale follow-up survey conducted by the social science survey center of Sun Yat-sen University, focusing on the current situation and changes of China's labor force. The sample covers 29 provinces and cities across the country (except Hong Kong, Macao, Taiwan, Tibet and Hainan). The survey covers multiple research topics such as education, work, economic activities and social participation. At the same time, the multi-stage and multi-level probability sampling method is adopted. This provides good data support for the research of this paper.

This paper focuses on the impact of URRBMI on migrant workers' overwork. Therefore, this paper first identifies the migrant workers. Refer to the research of Wu et al. (23). The sample with registered residence as agricultural and half a year away from the place of residence is retained. At the same time, the samples whose occupation type is “farming” are excluded, with 4,956 samples retained. Secondly, in order to improve the rigor of the study, samples with different types of insurance participation and household registration were deleted during data processing. Panel data were obtained for two years after screening, and a total of 3,715 eligible samples were selected. After excluding the missing values of important variables and invalid samples, a total of 3,087 valid samples were obtained.

### Variable Selection and Descriptive Statistics

#### Explained Variable

Overwork refers to the overtime and over intensity labor behavior of workers in their work. Compared with the labor intensity and fatigue state of migrant workers, it is easier to obtain labor time. Therefore, this paper uses the commonly used indicator labor time in academic circles to measure whether migrant workers are overworked (24). Based on the limitation of labor time in the labor law of the people's Republic of China, this paper takes whether the weekly labor time exceeds 50 h and whether the weekly labor time exceeds 60 h as the identification criteria, and whether the weekly labor time exceeds 60 h is the core index of the later analysis (1).

According to the “Working hours in the past week” in CLDS personal questionnaire, this paper makes descriptive statistics on the Overwork status of migrant workers in 2016 and 2018, as shown in Table 1. In the whole sample, the average weekly working hours of migrant workers are 51.09 h, far more than the weekly working hours of workers stipulated in China's labor law shall not exceed 44 hours. At the same time, the phenomenon of migrant workers' overwork is relatively serious. Under the standard of 50 h of working time per week, the proportion of migrant workers' overwork is as high as 66.00%, while under the standard of 60 h of working time per week, 48.04% of migrant workers are overworked. The average overwork time per week is 15.43 h, indicating that the overworked migrant workers work overtime three times a week for at least 5 h each time. From different periods, the proportion of migrant workers in 2018 decreased compared with that in 2016, and the weekly average working hours decreased, but the excessive working hours increased slightly, indicating that the degree of excessive labor of migrant workers was deepened.

**Table 1:** Descriptive statistics of changes in migrant workers' overwork.

**Year**	**Proportion of excessive labor** **(50 h)**	**Proportion of excessive labor** **(60 h)**	**Weekly working hours**	**Overwork time**
2016	67.23%	48.28%	51.11 (hours)	14.43 (hours)
2018	48.28%	47.82%	51.08 (hours)	15.84 (hours)
Full sample	66.00%	48.04%	51.09 (hours)	15.144 (hours)

#### Explanatory Variables and Control Variables

**Figure 2:**
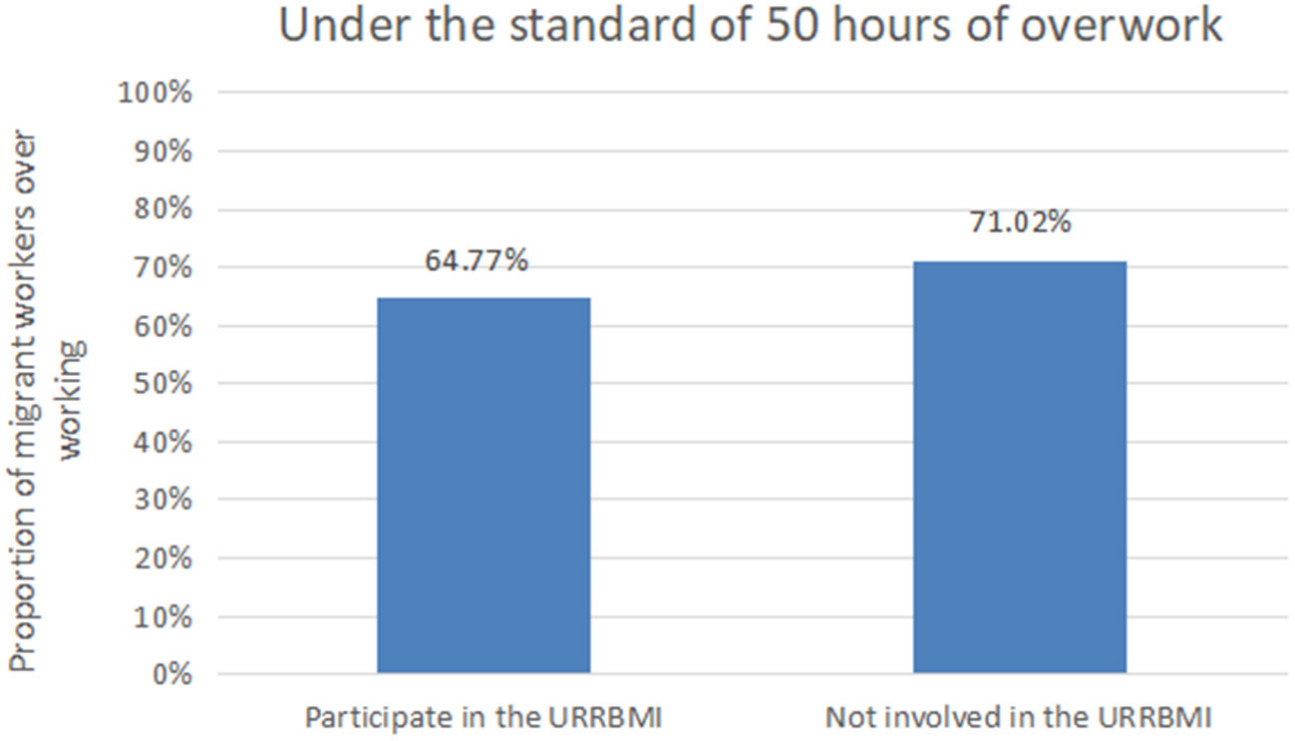
Proportion of migrant workers' overwork under 50 h standard.

**Figure 3:**
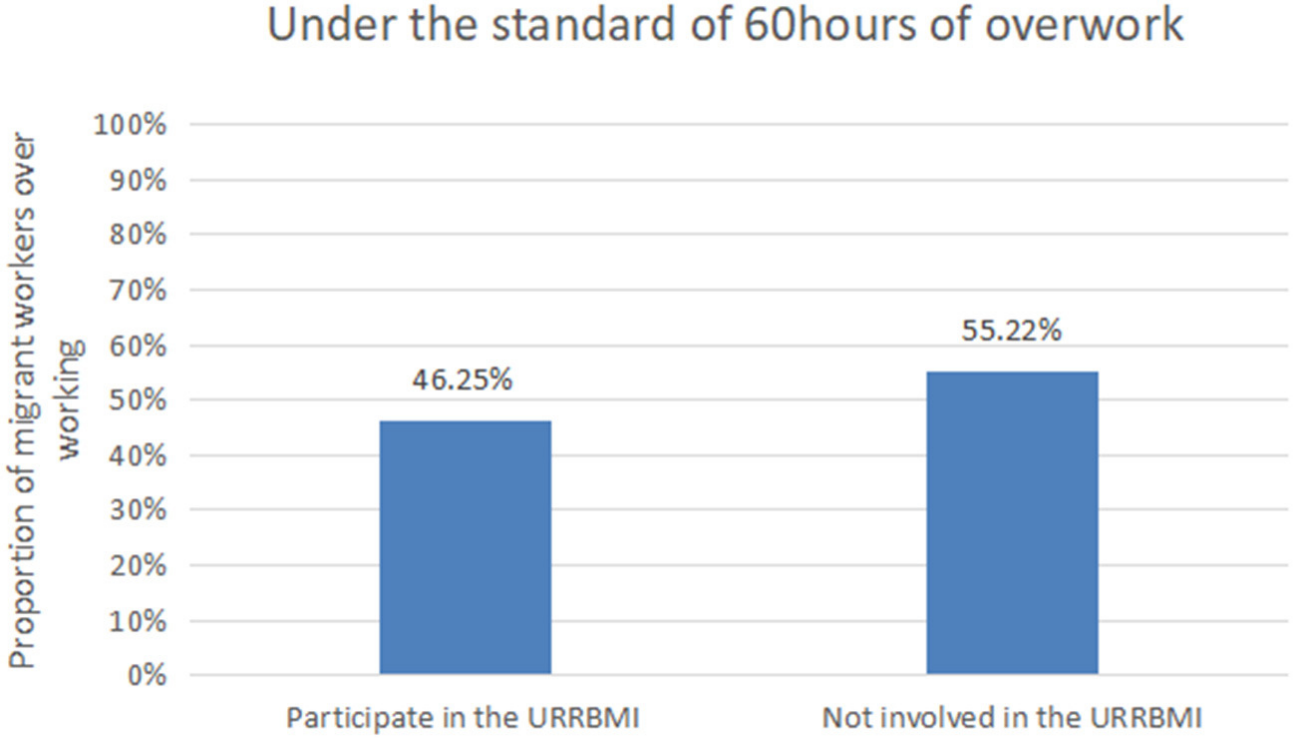
Proportion of migrant workers' overwork under 60 h standard.

According to the CLDS questionnaire, the investigator asked the respondents “Do you participate in urban and rural medical insurance?” to judge whether the area where migrant workers are located has implemented the URRBMI. If the respondent is insured for URRBMI, it is considered that the URRBMI has been implemented in the region, and the variable is assigned as 1. If the respondent is insured for NCMS, it is considered that the URRBMI has not been implemented in the region, and the variable is assigned as 0. Figures 2, 3, respectively show the relationship between the URRBMI and the proportion of migrant workers' overwork under different overwork standards. As can be seen from the figure, under the 50 h standard, the proportion of migrant workers participating in URRBMI was 67.77%, while the proportion of migrant workers not participating in URRBMI increased significantly, reaching 71.02%. Similarly, under the 60 h standard, the proportion of migrant workers who did not participate in URRBMI was greater than that of migrant workers who participated in URRBMI, reaching 55.22%. It can be seen that the implementation of URRBMI has alleviated migrant workers' excessive labor to a certain extent. In addition, by combing the existing studies, this paper adds some control variables that may affect migrant workers' excessive labor, as shown in Table 2.

**Table 2:** Definition and value of each variable.

**Variable Name**		**Variable Definition**	**Mean**	**Std. Err**.
Dependent variables	Overwork			
	Under 50 h standard	The weekly labor time exceeds 50 h = 1;Not exceeding = 0	0.660	0.474
	Under 60 h standard	The weekly labor time exceeds 60 h = 1;Not exceeding = 0	0.480	0.480
	Weekly working hours	Working hours in the past week	51.093	21.016
Independent variables	URRBMI	The sample area implements the URRBMI = 1;unenforced = 0	0.796	0.403
Control variables	Age	Age of respondents (years)	44.177	11.757
	Nation	Han nationality = 1;Other nationalities = 0	0.953	0.212
	Gender	Male = 1;female = 0	0.506	0.500
	Education level	0 = Never went to school;6 = primary school;9 = junior middle school;12 = High school / technical school / technical secondary school / vocational school;15 = junior college;16 = undergraduate;19 = master;23 = doctor	8.454	3.511
	level of health	1 = Very unhealthy;2 = Relatively unhealthy;3 = commonly;4 = Relatively healthy;5 = Very healthy	3.681	0.990
	Marital status			
	Unmarried	Unmarried = 1;other = 0	0.057	0.232
	Married	Married = 1;other = 0	0.913	0.286
	Divorced or widowed	Divorced or widowed = 1;other = 0	0.030	0.170
	Professional types	Private enterprise = 1;Individual business = 2 Enterprise of other nature = 3	1.604	0.901
	Monthly income	Natural logarithm of migrant workers' income in the past month	7.702	0.875
	Economic satisfaction	1 = Very dissatisfied;2 = Quite dissatisfied;3 = commonly;4 = Quite satisfied;5 = Very satisfied	3.207	1.029
	Family size	Number of family members living together	4.466	1.825
	Household consumption expenditure	Natural logarithm of total household consumption in the past year	10.513	0.867
	Toilet type	1 = Indoor;2 = Outdoor flushing toilet;3 = Outdoor non-flushing public toilet;4 = Outdoor non-flush toilet;5 = other	1.648	1.174
	Air pollution degree	1 = Very serious;2 = Relatively serious;3 = Not too serious;4 = It's not serious at all	3.016	0.873

### Model Selection

#### Benchmark Regression

When examining the impact of URRBMI on migrant workers' overwork, it will be affected by many unobservable factors. In order to eliminate the interference of other factors, this paper regards the implementation of the policy of URRBMI as a quasi natural experiment, and compares the differences of excessive labor changes between the groups impacted by the policy (treatment group) and the groups not impacted by the policy (control group), which is taken as the net effect of the policy. It is worth noting that URRBMI has obvious characteristics of pilot and gradual implementation, and there are differences in the time of URRBMI in different regions. Therefore, based on the study of Chang et al. (25), this paper judges whether URRBMI is implemented in this region according to the insurance type of the insured, that is, if the respondents participated in the medical insurance for urban and rural residents, the region was considered to have implemented URRBMI. Specifically, take the samples participating in NCMS in 2016 and 2108 as the control group, and the samples participating in NCMS in 2016 and URRBMI in 2018 as the experimental group, so as to establish a double difference model:


(1) $$\begin{array}{r} overwork_{ict}=\alpha_{0}+\alpha_{1}D_{ct}\times T_{i}+\alpha_{2}N_{ict}+\omega_{c}+\delta_{t}+\varepsilon_{ict} \end{array}$$

Among them, *i, c* and *t* respectively represent the surveyed migrant workers, the region where the sample is located and the visit time. The explained variable *overwork*_*ict*_indicates whether migrant workers *i* in City *c* are overworked at *t*. If migrant workers are overworked, *overwork*_*ict*_=1, Otherwise *overwork*_*ict*_=0. The key explanatory variable*D*_*ct*_of this paper indicates whether City *c* has implemented the URRBMI at *t*. If City *c* has implemented the URRBMI at *t*, then the pilot year *t* and subsequent years are assigned as 1, otherwise it is 0. *N*_*ict*_is the vector set of a series of control variables,ω_*c*_representing regional fixed effect,δ_*t*_represents time fixed effect. The coefficient [[Inline Image]]of [[Inline Image]]is the double difference estimator concerned in this paper, which reflects the impact of URRBMI on migrant workers' overwork.

#### Path Analysis

In order to empirically test the impact path proposed on the theoretical basis above, the following intermediary effect model is constructed:


(2) $$\begin{array}{rcl} overwork_{ict} & = & \alpha_{0}+\alpha_{1}D_{ct}\times T_{i}+\alpha_{2}N_{ict}+\omega_{c}+\delta_{t}+\varepsilon_{ict1} \end{array}$$

(3) $$\begin{array}{rcl} M_{ict} & = & \lambda_{0}+\lambda_{1}D_{ct}\times T_{i}+\lambda_{2}N_{ict}+\omega_{c}+\delta_{t}+\varepsilon_{ict2} \end{array}$$

(4) $$\begin{array}{l} overwork_{ict}=\beta_{0}+\beta_{1}D_{ct}\times T_{i}+\beta_{2}M_{ict}+\beta_{3}N_{ict}+\omega_{c}\\ \;+\delta_{t}+\varepsilon_{ict3} \end{array}$$

The test ideas of mediation effect model are as follows: firstly, the model setting of equation (2) is consistent with that of equation (1), and equations (3) and (4) are further estimated based on the significant coefficient α_1_of variable *D*_*ct*_ × *T*_*i*_. *M*_*ict*_is the intermediary variable of this paper. If the coefficient λ_1_of equation (3) and the coefficient β_2_of equation (4) are significant, it shows that the URRBMI has a significant impact on migrant workers' overwork through the intermediary variable. The above equation and the benchmark equation also use double difference to estimate relevant parameters. If the coefficient β_1_of equation (4) is significant, it indicates that it belongs to partial intermediary. If the coefficient β_1_of equation (4) is not significant, it is complete intermediary.

## Results

### Benchmark Regression

Firstly, this paper uses the binary choice model probit to estimate the impact of URRBMI on migrant workers' overwork. Considering that the regression results are only statistically significant in significance and impact direction, Table 3 reports the average marginal effect of each variable on the impact of migrant workers' overwork. It can be seen from Table 3 that whether the weekly working hours of migrant workers exceed the 50 h standard or the 60 h standard, the URRBMI can significantly alleviate the phenomenon of migrant workers' overwork, especially under the 60 h standard, the mitigation effect of URRBMI on migrant workers' overwork is more significant. Specifically, under the 50 h standard, the impact of URRBMI on migrant workers' overwork is significantly negative at the level of 5%, indicating that participating in URRBMI reduces the probability of migrant workers' overwork by 7%. Under the 60 h standard, URRBMI can also significantly reduce the probability of migrant workers' overwork. In addition to the analysis of whether overwork is used as the explained variable, this paper further takes the labor time of migrant workers as the explained variable, and uses the fixed effect empirical test to test the impact of the URRBMI on the labor time of migrant workers. Table 3 shows that the URRBMI can reduce the weekly working hours of migrant workers by 2.575 h, which is significant at the level of 10%. This is consistent with the above estimation results, which shows that the URRBMI can not only significantly reduce the labor time of migrant workers, but also alleviate the excessive labor of migrant workers.

**Table 3:** Benchmark regression.

**Variable**	**Overwork (50 h)**	**Overwork (60 h)**	**Working hours**	**Overwork (50 h)**	**Overwork (60 h)**	**Working hours**
URRBMI	−0.070**	−0.073**	−2.575*			
	(0.032)	(0.030)	(1.383)			
URRBMI × year				−0.127**	−0.144***	−8.901***
				(0.050)	(0.044)	(2.047)
Education level	−0.032***	−0.022***	−0.817***	−0.032***	−0.021***	−0.789***
	(0.003)	(0.003)	(0.134)	(0.003)	(0.003)	(0.134)
Age	−0.004***	−0.005***	−0.180***	−0.003***	−0.005***	0.176***
	(0.001)	(0.001)	(0.043)	(0.001)	(0.001)	(0.043)
Healthy	−0.004	−0.028**	−0.415	−0.003	−0.027**	−0.466
	(0.011)	(0.010)	(0.475)	(0.011)	(0.010)	(0.472)
Monthly income logarithm	0.045***	0.019*	2.384***	0.049***	0.020*	2.380***
	(0.012)	(0.011)	(0.508)	(0.012)	(0.011)	(0.505)
Toilet type	0.005	0.004	−0.980**	0.001	0.004	−1.027**
	(0.011)	(0.010)	(0.461)	(0.011)	(0.010)	(0.460)
Economic satisfaction	−0.017*	−0.010	−0.652*	−0.019**	−0.011	−0.638
	(0.009)	(0.009)	(0.395)	(0.009)	(0.009)	(0.393)
Air pollution degree	−0.001	−0.006	−0.552	−0.001	−0.006	−0.484
	(0.011)	(0.010)	(0.462)	(0.011)	(0.010)	(0.460)
Household consumption expenditure	−0.029 **	−0.010	−0.670	−0.028**	−0.010	−0.627
	(0.012)	(0.011)	(0.499)	(0.012)	(0.011)	(0.498)
Nation	−0.051	−0.037	−3.868*	−0.050	−0.037	−3.871*
	(0.049)	(0.045)	(2.037)	(0.049)	(0.045)	(2.035)
Married	−0.017	0.010	−0.778	−0.023	0.012	−0.704
	(0.039)	(0.037)	(1.699)	(0.039)	(0.037)	(1.689)
Divorced or widowed	−0.042	0.005	0.320	−0.034	0.013	0.376
	(0.074)	(0.071)	(3.204)	(0.075)	(0.072)	(1.690)
Gender	0.065***	0.028	0.960	0.062***	0.026	0.776
	(0.019)	(0.018)	(0.823)	(0.019)	(0.018)	(0.820)
Family size	0.011**	−0.000	0.173	0.010*	−0.006	0.110
	(0.005)	(0.000)	(0.231)	(0.005)	(0.005)	(0.230)
Professional types	0.010	0.034***	−0.042	0.112	0.041***	0.153
	(0.010)	(0.009)	(0.438)	(0.010)	(0.009)	(0.153)
Control variables	controlled	controlled	controlled	controlled	controlled	controlled
*Pseudo R^2^/R^2^*	0.0582	0.0448	0.0522	0.0568	0.0444	0.0539
Sample size	3087	3087	3087	3087	3087	3087

Standard errors are in parentheses; ^*^, ^**^ and ^***^ means passing the test at the significance levels of 10%, 5%, and 1%, respectively. Except for labor time, all other reports are probit marginal effect.

Secondly, according to the setting of the above double difference model, this paper uses the double difference model to further investigate the impact of URRBMI on migrant workers' overwork, which is also the core method of the empirical test. Table 3 results show that taking the 60 h standard as an example, the impact of the cross term of URRBMI and year on migrant workers' overwork is significant at the level of 1%, with a coefficient of −0.144, indicating that participating in URRBMI will significantly reduce the probability of migrant workers' overwork. From the results of labor time regression equation, the coefficient of double difference is −8.901, which is significant at the level of 1%, indicating that participating in URRBMI will significantly reduce the labor time of migrant workers and reduce their weekly labor time by about 8.9 h. Compared with the previous probit regression results, the negative impact of URRBMI on migrant workers' overwork and working time is significantly increased. To sum up, the URRBMI has significantly reduced the labor time of migrant workers, reduced their probability of overwork, and improved the welfare of migrant workers.

### Robustness Check

Based on the previous empirical analysis, it is found that the URRBMI can significantly alleviate migrant workers' overwork. In order to reduce the error and ensure the robustness of the results, this paper uses PSM-DID model to re estimate. The most important and key premise for the application of the double difference method: the control group and the control group must meet the common trend hypothesis, that is, if they are not affected by the URRBMI, there is no systematic difference over time in migrant workers' overwork. However, in view of the actual situation of migrant workers' excessive labor, there are still endogenous problems caused by sample selection errors, which is difficult to meet the common trend hypothesis. In this paper, the double differential propensity score matching method (PSM-DID) proposed by Heckman et al. (26) can effectively alleviate this problem. The basic idea is to match the individuals with similar propensity scores in the control group, remove the selective errors caused by non-randomness, so that the screened samples are only different in terms of excessive labor, and other characteristic variables are as similar as possible, so as to obtain the net effect of the URRBMI on the impact of excessive labor of migrant workers.

**Figure 4:**
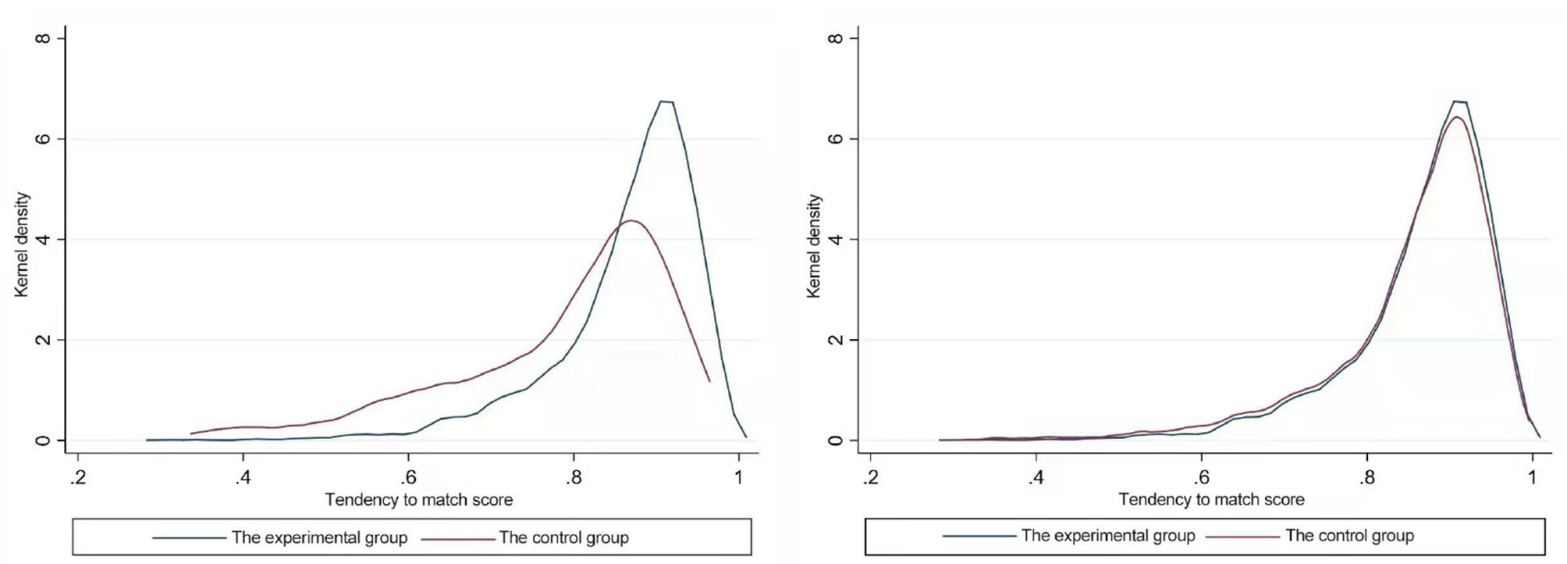
Kernel density function before and after propensity score matching of urban-rural medical insurance integration.

It should be noted that in order to ensure the effectiveness of propensity score matching, we first need to test the common support and balance hypothesis. Figure 4 is a test result of the common support hypothesis using the kernel density function method. From the nuclear density function diagram before matching, it can be seen that there are obvious differences in the nuclear density function curves between the experimental group and the control group, and there are enough overlaps, indicating that they meet the common support hypothesis. The nuclear density function curves of the matched experimental group and the control group are in good agreement, indicating that the test of the balance hypothesis is satisfied after the propensity score matching. Table 4 further proves that there is no significant difference in covariate characteristics between the two groups after matching, indicating that PSM model is reasonable.

**Table 4:** Balance test results.

**Variable**	**Sample**	**Mean**		**Standardization deviation**		**T statistic**
		**Control group**	**Treatment group**	**Standardization deviation**	**Bias%**	
Education level	Before matching	8.648	9.079	14.0	96.9	2.63
	After matching	8.958	9.079	−0.4		−0.16
Age	Before matching	42.28	42.168	−1.1	−400.5	−0.20
	After matching	41.59	42.168	5.4		1.97
Healthy	Before matching	3.812	3.838	3.0	−51.4	0.59
	After matching	3.878	3.838	−4.5		−1.66
Monthly income logarithm	Before matching	7.488	7.752	29.7	93.8	6.02
	After matching	7.736	7.752	1.8		0.70
Toilet type	Before matching	2.062	1.493	−46.3	96.1	−10.15
	After matching	1.471	1.493	1.8		0.77
Economic satisfaction	Before matching	3.201	3.207	0.5	−32.9	0.10
	After matching	3.200	3.207	0.7		0.25
Air pollution degree	Before matching	3.181	2.920	−30.7	97.8	−5.93
	After matching	2.926	2.920	−0.7		−0.24
Household consumption expenditure	Before matching	10.4	10.586	23.9	74.8	4.46
	After matching	10.539	10.586	6.0		2.17
Nation	Before matching	0.905	0.962	23.3	73.6	5.42
	After matching	0.947	0.962	6.1		2.65
Married	Before matching	0.949	0.910	−15.4	49.9	−2.79
	After matching	0.890	0.910	7.7		2.39
Divorce or widowhood	Before matching	0.015	0.019	2.9	−76.9	0.56
	After matching	0.013	0.019	5.2		1.98
Gender	Before matching	0.613	0.571	−8.4	91.8	−1.65
	After matching	0.575	0.571	−0.7		−0.25
Professional types	Before matching	1.752	1.565	−20.5	71.6	−4.13
	After matching	1.617	1.565	−5.8		−2.17
Family size	Before matching	4.527	4.410	−6.6	−17.3	−1.26
	After matching	4.274	4.410	7.7		2.82
Year	Before matching	2017.1	2017	−5.3	9.0	−1.05
	After matching	2017	2017	4.9		1.76
Province	Before matching	2.000	1.600	16.6	77.9	3.16
	After matching	2.000	2.100	−3.7		−1.29

Table 4 reports the balance test results of propensity score matching. It can be seen from Table 4 that after propensity score matching, the distribution of covariates in the treatment group and the control group has been relatively balanced, and the difference is no longer significant. Specifically, the absolute value of covariate standardization deviation in the matched treatment group and control group is <10%. The results of *t*-test also show that there is no significant difference between the two groups. Therefore, it can be considered that the sample data in this paper are suitable for estimation by PSM-DID method, and the conclusion is reliable.

After the common support and balance hypothesis test, this paper makes a double difference on the matched samples. Results as shown in Table 5, the URRBMI can still significantly alleviate the excessive labor of migrant workers under either the 50 h standard or the 60 h standard, and its impact coefficient is slightly higher than the benchmark estimation results above. In terms of working hours, after migrant workers participate in the URRBMI, their weekly working hours are reduced by about 8.66 h. To sum up, the URRBMI can significantly reduce the working hours of migrant workers and alleviate their overwork.

**Table 5:** Double differential propensity score matching (PSM-DID).

**Variable**	**Overwork**	**Overwork**	**Working**
	**(50 h standard)**	**(60 h standard)**	**hours**
URRBMI × Year	−0.145***	−0.151***	−8.658***
	(0.051)	(0.045)	(2.194)
Control variables	Controlled	Controlled	Controlled
*Pseudo R^2^/R^2^*	0.061	0.052	0.057
Sample size	3,012	3,012	3,012

Standard errors are in parentheses; ^*^, ^**^ and ^***^ means passing the test at the significance levels of 10%, 5%, and 1%, respectively. Except for labor time, all other reports are probit marginal effect.

### Heterogeneity Analysis

#### Migrant Workers of Different Generations

Previous studies have shown that age is an important factor affecting migrant workers' overwork. There are significant differences between the old generation of migrant workers and the new generation of migrant workers in education level, cultural pursuit and economic pressure, which will directly affect whether they overwork. Therefore, according to the year of birth, this paper takes the samples born after 1980 as the new generation of migrant workers, and those born before 1980 are divided into the old generation of migrant workers, and then estimates the impact of URRBMI on the Overwork of migrant workers in the two sub samples. Results as shown in Table 6, the URRBMI has significantly reduced the Overwork probability of the older generation of migrant workers by 22.5 and 17.6%, and reduced their weekly working hours by about 9.03 h, both significantly at the level of 1%. The URRBMI has no significant impact on the overwork and working time of the new generation of migrant workers.

**Table 6:** Heterogeneity analysis.

**Different generations**	**Older generation of migrant workers**			**New generation of migrant workers**		
**Variable**	**Overwork (50 h)**	**Overwork (60 h)**	**Working hours**	**Overwork (50 h)**	**Overwork (60 h)**	**Working hours**
URRBMI × year	−0.225***	−0.176***	−9.025***	0.097	0.006	−3.023
	(0.062)	(0.052)	(3.001)	(0.110)	(0.101)	(6.267)
Constant term			−23.515			165.142
			(71.655)			(102.246)
Control variable	Controlled	Controlled	Controlled	Controlled	Controlled	Controlled
Sample size	2206	2206	2206	881	881	881
*Pseudo R^2^/R^2^*	0.058	0.049	0.074	0.104	0.094	0.181
**Different regions**	**Eastern region**			**Central and Western regions**		
**Variable**	**Overwork (50 h)**	**Overwork (60 h)**	**Working hours**	**Overwork (50 h)**	**Overwork (60 h)**	**Working hours**
URRBMI × year	−0.091	−0.087	−7.884*	−0.193***	−0.210***	−15.928**
	(0.081)	(0.068)	(4.238)	(0.060)	(0.059)	(5.400)
Constant term			−14.055			219.672
			(37.446)			(162.790)
Control variable	Controlled	Controlled	Controlled	Controlled	Controlled	Controlled
Sample size	2,074	2,074	2,074	1,013	1,013	1,013
*Pseudo R^2^/R^2^*	0.085	0.042	0.061	0.075	0.085	0.156
**Different industries**	**The second industry**			**The third industry**		
**Variable**	**Overwork (50 h)**	**Overwork (60 h)**	**Working hours**	**Overwork (50 h)**	**Overwork (60 h)**	**Working hours**
URRBMI × year	−0.182**	−0.540**	−10.506**	−0.150**	−0.230***	−8.293**
	(0.076)	(0.224)	(4.758)	(0.068)	(0.078)	(3.140)
Constant term			76.654			61.846
			(108.162)			(14.901)
Control variable	Controlled	Controlled	Controlled	Controlled	Controlled	Controlled
Sample size	2,037	2,037	2,037	1,050	1,050	1,050
*Pseudo R^2^/R^2^*	0.052	0.069	0.073	0.079	0.064	0.148
**Different levels of education**	**Junior high school the following**			**Junior high school above**		
**Variable**	**Overwork (50 h)**	**Overwork (60 h)**	**Working hours**	**Overwork (50 h)**	**Overwork (60 h)**	**Working hours**
URRBMI × year	−0.131**	−0.142***	−7.148**	−0.209**	−0.155*	−13.120**
	(0.062)	(0.053)	(3.487)	(0.100)	(0.087)	(6.481)
Constant term			3.096			−74.103
			(85.668)			(124.297)
Control variable	Controlled	Controlled	Controlled	Controlled	Controlled	Controlled
Sample size	2,315	2,315	2,315	772	772	772
*Pseudo R^2^/R^2^*	0.078	0.059	0.108	0.070	0.074	0.235

Standard errors are in parentheses; ^*^, ^**^ and ^***^ means passing the test at the significance levels of 10%, 5%, and 1%, respectively. Except for labor time, all other reports are probit marginal effect.

#### Migrant Workers in Different Regions

According to the respondents' region, this paper divides all samples into eastern, central and western regions to test the impact of URRBMI on migrant workers' overwork in different regions. Results as shown in Table 6, the URRBMI has a significant negative impact on the overwork and working time of migrant workers in the central and western regions. Taking the 60 h standard as an example, migrant workers in the central and western regions participated in URRBMI, which reduced the probability of overwork by 21% and the weekly working hours by 15.93 h, which was significant at the level of 1%. For migrant workers in the eastern region, the URRBMI can only reduce their weekly working hours by 7.88 h, which is significant at the level of 10%, but the impact on migrant workers' overwork in the eastern region is not significant.

#### Migrant Workers in Different Industries

According to the industry type of respondents, the sample of migrant workers engaged in mining, manufacturing and construction will be taken as the secondary industry, and the samples engaged in wholesale and retail trade, catering and other industries are divided into migrant workers engaged in the tertiary industry. Then this paper estimates the impact of URRBMI policy on the overwork of migrant workers in different industries. As shown in Table 6, URRBMI policies can significantly reduce the probability of overwork of migrant workers in different industries. Specifically, URRBMI policy significantly reduced the probability of overwork of migrant workers in the secondary industry by 18.2 and 54.0% respectively, and the weekly working hours were reduced by about 10.51 h, both significantly at the level of 5%. URRBMI policy also significantly reduced the probability of overwork of rural migrant workers in the tertiary industry by 15.0 and 23.0% respectively, and reduced the weekly working hours by about 8.29 h.

#### Migrant Workers With Different Levels of Education

Referring to Susanna et al. (27) research, this paper divides all migrant workers into samples with high education level and low education level according to whether they have received junior high school education or not, and tests the influence of URRBMI policy on overwork of migrant workers with different education levels. The results are shown in Table 6, URRBMI policy can significantly reduce the probability of overwork of migrant workers with high education levels by 20.9 and 15.5% respectively, and weekly working hours by about 13.12 h. For migrant workers with low education levels, the URRBMI policy also significantly reduced the probability of overwork by 13.1 and 14.2% respectively, and reduced weekly working hours by about 7.15 h.

## Impact Path Analysis

According to the previous analysis of the impact mechanism, this paper expects that the URRBMI may affect the Overwork of migrant workers through the following two paths: First, the URRBMI can effectively reduce the probability of migrant workers' overwork by improving their sense of social integration. Identity is the degree to which people accept their status, roles, images and relationships with others in society (28). As a special group under the arrangement of the household registration system, migrant workers in China are subject to institutional discrimination due to their departure from their occupation and identity, which leads to the dilemma of their identity. Fair and reasonable design of political system and distribution of public rights have become important ways to improve the identity of migrant workers (29). Therefore, in this paper, the questionnaire “whether you participated in the last neighborhood committee vote” is used as the measurement index of social identity, no vote is assigned 0, and participation in voting is assigned 1. Second, the URRBMI will reduce the time of overwork by improving the health status of migrant workers. The health status variable is measured by “Have you had physical pain in the past month?” in the personal questionnaire. According to the model set above, this part tests the above influence paths step by step according to the mediation effect causal step method (30).

Table 7 shows the regression results of the intermediary effect model constructed with “identity” and “health level” as intermediary variables. Among them, the cross item of URRBMI and year represents the net effect of URRBMI on migrant workers' overwork. As can be seen from Table 7, the impact coefficient of the cross item of URRBMI and year on migrant workers' identity is significant at the level of 5%, indicating that URRBMI helps to improve migrant workers' identity. After adding the intermediary variable “identity” into the regression equation, the impact coefficient of URRBMI on migrant workers' overwork is smaller than that without controlling the intermediary variable, and the impact of identity on migrant workers' overwork is significant at the level of 1%. It shows that the mitigation effect of URRBMI on migrant workers' overwork is partly through improving their sense of identity, which is basically consistent with the previous mechanism analysis. From the perspective of the impact mechanism of “health level,” the URRBMI plays a significant role in improving the health level of migrant workers at the 5% level. Similarly, after the intermediary variable “health level” is added to the regression equation, the impact of URRBMI and health level on migrant workers' overwork is significant at the 1% level, indicating that URRBMI alleviates migrant workers' overwork by improving migrant workers' health status. To sum up, the URRBMI reduces the probability of migrant workers' overwork by improving their social identity and health level.

**Table 7:** Impact path of URRBMI on migrant workers' overwork.

	**Overwork**	**Sense of identity**	**Level of health**	**Overwork**
URRBMI × year	−0.729***	0.156**	0.454**	−0.145***
	(0.223)	(0.067)	(0.229)	(0.045)
Sense of identity				−0.071***
				(0.194)
Level of health				−0.039***
				(0.014)
Control variables	Controlled	Controlled	Controlled	Controlled
Sample size	2,730	2,730	2,730	2,730
*Pseudo R^2^/R^2^*	0.053	0.127	0.097	0.057

Standard errors are in parentheses; ^*^, ^**^ and ^***^ means passing the test at the significance levels of 10%, 5%, and 1%, respectively. Except for labor time, all other reports are probit marginal effect.

## Discussion

The implementation of URRBMI policy has narrowed the difference between urban and rural medical security and provided institutional guarantee for protecting the health rights and interests of migrant workers. Firstly, this paper theoretically expounds the relationship between the URRBMI and migrant workers' excessive labor. At the same time, using the survey data of China labor force dynamic survey (CLDS), this paper empirically tests the impact of URRBMI on migrant workers' overwork. The results show that the URRBMI can significantly reduce the labor time of migrant workers and reduce the probability of overwork. Although previous studies did not directly evaluate the impact of the URRBMI on the labor supply of migrant workers, some studies tested the impact of urban workers' medical insurance and other forms of public medical care on migrant workers' excessive labor from the perspective of health rights and interests (6). The conclusion is consistent with the results of this paper. On the one hand, the URRBMI policy improves the sense of identity and social integration of migrant workers by eliminating the barriers to health rights and interests in urban and rural China, thus reducing their probability of overwork. On the other hand, the implementation of URRBMI policy enables migrant workers to enjoy the same medical services as urban residents, reduces their preventive labor supply, and improves their labor supply efficiency by improving their health conditions, so that migrant workers do not have to work overtime to increase their income.

Secondly, the heterogeneity analysis shows that the URRBMI can significantly alleviate the Overwork of the old generation of migrant workers, but has no significant impact on the new generation of migrant workers. Compared with the new generation of migrant workers, long-term high-intensity labor has seriously damaged the health of the older generation of migrant workers. Meanwhile, the physical function of the older generation of migrant workers is declining with the increase of age. The older generation of migrant workers are facing greater health risks, leading to the extension of working hours to obtain more income. The implementation of URRBMI policy can reduce the medical burden of the older generation of migrant workers, reduce the income fluctuation caused by health risks, and thus reduce their excessive working hours. Between different regions, Considering the obvious differences in the level of economic development, the proportion of medical insurance reimbursement and the acceptance of migrant population in different regions of China, the impact of URRBMI on migrant workers' overwork in different regions may be heterogeneous. The study found that the URRBMI can effectively alleviate the Overwork of migrant workers in the central and western regions, but it has no impact on the Overwork of migrant workers in the eastern region. The reason for this phenomenon is that the level of economic development in the eastern region is high, and the phenomenon of migrant workers working overtime is common in this region, resulting in the fact that the URRBMI does not significantly alleviate their excessive labor, but the labor intensity in the central and western regions is less than that in the eastern region, and there are fewer overtime in daily work, Therefore, when impacted by the policy of URRBMI, the probability of overwork decreases significantly (31).

In addition to the above heterogeneity, the impact of the URRBMI policy on the overwork of migrant workers will be heterogeneous due to the different industry types of migrant workers. It is found that the policy of URRBMI has a greater alleviating effect on migrant workers in the secondary industry than in the tertiary industry. The possible reason is that for migrant workers engaged in traditional secondary industries (such as construction and manufacturing), the relatively poor working environment and long-term heavy physical labor have seriously damaged their health status, making their health human capital far inferior to migrant workers engaged in the tertiary industry. At the same time, the education level of migrant workers in the secondary industry is generally not high, and the corresponding positions have strong substitution, which makes the vocational mobility of migrant workers in the tertiary industry greater, the wage level is generally low, and the lack of social security provided by employers, resulting in the phenomenon of excessive labor of migrant workers in the secondary industry more serious. Therefore, the URRBMI policy has significantly improved the medical accessibility and health status of migrant workers in the secondary industry, making the probability of overwork of migrant workers in the secondary industry more obvious. At the same time, this paper further tests the impact of the URRBMI policy on the overwork of migrant workers with different education levels. The results show that the URRBMI policies can significantly alleviate the overwork of migrant workers with different educational levels, and the effect on overwork of migrant workers with higher educational levels is more significant. The possible reason is that the higher the education level of migrant workers, the more they understand about the policy setting of URRBMI, and the better they can enjoy the welfare brought by the policy. Compared with migrant workers with low education level, they are not willing to spend more time and energy to participate in the URRBMI (32). Therefore, the URRBMI policy has a more significant effect on alleviating the overwork of migrant workers with high education level. Finally, there are few existing studies to explore the impact path of medical insurance on migrant workers' labor supply. Therefore, this paper further explores the impact of URRBMI on migrant workers' excessive labor from the perspectives of health level and identity. The results show that health level and identity play a partial intermediary role between the URRBMI and migrant workers' overwork.

## Conclusion

It is worth noting that the econometric method used in this paper can only reflect the impact of URRBMI on migrant workers' excessive labor from the sample as a whole, and determine the causal relationship between URRBMI and migrant workers' excessive labor. It is impossible to deeply explore whether each sample can alleviate its excessive labor after participating in URRBMI (33). In the future, we can further use the method of case analysis to analyze the individual differences of the impact of URRBMI on migrant workers' overwork. At the same time, due to the inconsistent implementation progress of the policy in various cities in China, there are differences in the integration mode, financing level and medical insurance treatment of the policy. However, limited by the survey data, it is difficult to carry out in-depth analysis from the details of the policy, and only the comprehensive impact of the overall planning policy can be analyzed. Therefore, the follow-up research can analyze the impact of URRBMI on migrant workers' labor supply from the perspective of different integration models, and reveal the deep-seated institutional factors behind it.

Most importantly, some policy implications can be derived from the empirical analysis. Migrant workers' long-term excessive labor behavior has seriously damaged their physical and mental health and reduced China's potential labor dividends. The implementation of URRBMI policy can significantly alleviate the excessive labor of migrant workers. Therefore, the government should accelerate the progress of medical insurance integration and expand the scope of medical insurance integration. At present Chinese health care has not been implemented nationwide transferring and carry, this is the main shackles of employment locking and work chains effect, only a completely dismantle free movement of Labor barriers and improve the efficiency of labor element configuration, to further promote the migrant workers' income, as much as possible to reduce the excessive labor possible. Second, improve the medical security system and strengthen the welfare protection of vulnerable groups. In particular, we should pay attention to coordinated regional development, give more preferential policies to regions with relatively backward economic development, and strengthen external financial support to narrow the gap between different regions. Finally, the popularization and publicity of public medical information should be strengthened to guide migrant workers to make reasonable diagnosis and treatment, so as to reduce migrant workers' blind dependence on medical treatment and the probability of unnecessary diagnosis and treatment, thus reducing migrant workers' medical expenses and improving their serious overwork situation.

## Data Availability Statement

The datasets China labor force dynamic survey (CLDS) presented in this article are not readily available because the ownership and copyright of the data belong to Sun Yat-sen University. The authors ZX and BL have applied to Sun Yat-sen University and have been approved. We have the right to use this database, but we have not obtained the ownership and copyright of the data, so we can't upload and publish this data. Requests to access the dataset should be directed to http://css.sysu.edu.cn. The data will be available after the application is approved.

## Author Contributions

ZX is responsible for research design, data processing, model design, and text writing. BL has made great contributions in the process of article conception and revision, including the improvement of analysis framework and the application of methods, and provided project support. Both authors contributed to the article and approved the submitted version.

## Funding

This study was supported by the Zhejiang Provincial Social Science Foundation of China under Grant No. 20NDQN299YB, the Zhejiang Provincial Natural Science Foundation of China under Grant No. LQ20G030004, and the Fundamental Research Funds for the Provincial Universities of Zhejiang under the Grant No. 2020YQ012.

## Conflict of Interest

The authors declare that the research was conducted in the absence of any commercial or financial relationships that could be construed as a potential conflict of interest.

## Publisher's Note

All claims expressed in this article are solely those of the authors and do not necessarily represent those of their affiliated organizations, or those of the publisher, the editors and the reviewers. Any product that may be evaluated in this article, or claim that may be made by its manufacturer, is not guaranteed or endorsed by the publisher.
